# Evaluating the sustainable traffic flow operational features of an exclusive spur dike U-turn lane design

**DOI:** 10.1371/journal.pone.0214759

**Published:** 2019-04-10

**Authors:** Yang Shao, Xueyan Han, Huan Wu, Huimin Shan, Shaowei Yang, Christian G. Claudel

**Affiliations:** 1 Traffic and Road Engineering Center, Highway Academy, Chang’an University, Xi’an, Shaanxi, China; 2 Xi’an Shiyou University, Xi’an, Shaanxi, China; 3 University of Texas at Austin, Austin, Texas, United States of America; University of British Columbia, CANADA

## Abstract

The traditional U-turn design has significantly improved traffic operations for relieving traffic congestion. However, the U-turn diversion and merge segments still cause traffic conflicts and delays. In this paper, an exclusive spur dike U-turn lane (ESUL) is proposed with the aim of addressing the disadvantages of the traditional U-turn design. ESUL provides a separate U-turn lane to diverge, decelerate, U-turn, accelerate and merge without interacting with through traffic. The effectiveness of ESUL is demonstrated through a field data investigation, simulation and analysis with VISSIM software. The proposed design is evaluated in terms of three parameters: travel time, delay and number of stops. Compared to the traditional U-turn design, ESUL can reduce travel time by 29.15%, delay by 66.70% and the number of stops by 100% at most. The results showed that ESUL has better performance than the traditional U-turn design and could be implemented to reduce traffic congestion and the potential hazards caused by U-turn maneuvers.

## Introduction

U-turn traffic has long been regarded as a dilemma. Establishing a U-turn opening could cause some traffic congestion, whereas forbidding U-turns will cause vehicles that need to perform a U-turn detour a long distance. U-turn designs are mainly used under two circumstances: the first case is making a U-turn rather than a direct left turn (DLT), and the second case is for when vehicles need to make a U-turn. However, some traffic conflicts, delay and stops still exist even with a U-turn design. To reduce such problems, many alternative measures have been proposed to improve the performance of U-turn designs, such as signalization [1, 2], exclusive left turn lane design [3, 4] and some novel techniques such as autonomous vehicles [5, 6]. Among these measures, many different solutions have specific application conditions and restrictions, and the U-turn issue still has potential for improvement. The facilities design is still a significant way from solving existing problems.

Many studies on U-turn designs have been conducted over the past decades. Median U-turn intersection treatment has been considered as an alternative measure to reduce congestion and traffic conflicts at intersection areas [7, 8]. U-turn curves installed for improving traffic flow at busy intersections produce their desired effects only when there is minimal interaction between cars [9, 10]. Separation distances significantly impact the safety of the street segments between driveways and downstream U-turn locations [11, 12]. A dual-bay design with different turning radii for small and large vehicles for U-turning vehicles can significantly improve the operations at intersection areas, particularly when the volume/capacity ratio is small and the ratio of left-turning to through traffic is small [13]. The disordered U-turning vehicles will delay transportation, cause more traffic jams, consume more fuel, and produce more air pollution [14, 15]. The vehicles that want to make a U-turn must travel in the inner lane after changing lanes at a sufficient distance from upstream. In China, lane changing is a transient behavior that randomly occurs with high frequency, which is the main feature of aggressive driving. The influence of lane changing on local traffic persists for approximately 15 to 30 s, with the influence rapidly increasing and then slowly declining. Lane changing behavior will increase the risk of high-speed car-following behavior [16, 17]. Making U-turns rather than using traffic lights for left turn flow could improve safety in crashes [18].

U-turn designs also have a relationship with DLT designs. Various left turn designs have been used in urban intersections to reduce problems accompanying DLT vehicles [19, 20]. The difficulty of completing a left turn is evident in crash statistics, which indicate that 45 percent of all crashes that occur at intersections throughout the United States involve left-turning vehicles, even though left-turning movements represent a disproportionately small percentage (10-15 percent) of all approaching traffic [21, 22]. DLTs from main streets or branch roads are prohibited by the use of a noncrossing median and/or directional median openings [23]. Vehicles turning left will be guided to detour downstream to a U-turn location rather than make a left turn [24]. Superstreet intersections, crossover displaced left turn intersections, and upstream signalized crossover schemes are common U-turn designs as median U-turn intersections (MUTIs) [25, 26].

**Fig 1:**
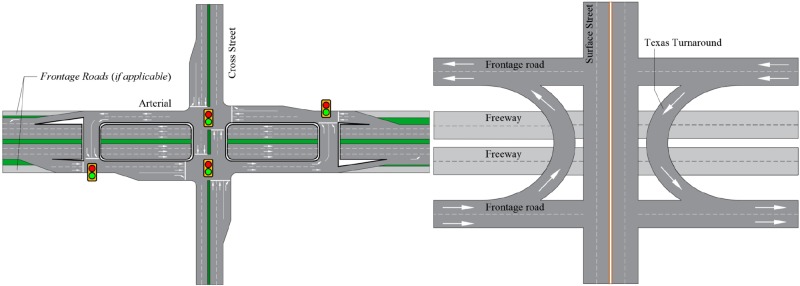
Examples of U-turn design applications. a:Michigan U-turn Design. b:Texas U-turn Design.

MUTI, an known as the Michigan U-turn, is the most famous and widely used U-turn design for vehicles [27, 28]. The geometric design of MUTI is shown in Fig 1. Several principles should be followed during the design process: (1) separate the left-turning vehicle and through traffic in opposite directions to reduce conflicts; (2) control the left turn signal phase; and (3) reduce and/or keep the efficiency of other movements of the area. The previous studies and applications of MUTI have demonstrated that MUTI provides a significant improvement in terms of reducing conflicts and delays and improves the safety situation at intersection areas [29, 30]. However, MUTI has a significant limitation that restricts its application around the world, namely, MUTI requires a wide enough median for vehicles to perform a U-turn. Small vehicles (cars) and large vehicles (buses/trucks) have different turning radii. Large vehicles require a much larger median width. It is very difficult to apply MUTI in an intersection with a narrow median [30]. Similar to MUTI, the Texas U-turn, also known as the Texas turnaround, boomerang, or loop around, is a lane that allows cars to travel on one side of a one-way frontage road to perform a U-turn onto the opposite frontage road (typically crossing over or under a freeway or expressway). The U-turn designs are typically controlled by yield signs, which allow U-turning traffic to bypass two traffic signals and avoid crossing the local traffic twice.Both U-turn designs are shown in Fig 1.

Many of the previous studies have provided a median U-turn opening, focusing on the layout and location of the opening, as well as on the impact on the signal plan of the intersection. The width of the median is typically determined according to the turning radii of the U-turning vehicles, which limits the width of the median U-turn opening. In the case of a large number of U-turning vehicles, the queue length is larger than the width of the median U-turn opening, which will cause the U-turning vehicles to line up and result in the through vehicles having to wait, change lanes and/or even have accidents [31, 32]. The directions of U-turning vehicles and through vehicles generally have a large angle, which will cause a traffic conflict when U-turning vehicles need to merge. The proportion of vehicles complying with the yield rule is low in China, and the negative effect caused by U-turn conflicts is serious [33, 34]. Therefore, the U-turning vehicles and through vehicles should be channeled to drive separately, which will eliminate the harmful influence.

By separating U-turning and through vehicles far from the U-turn opening, no influence exists during the U-turn movement. Accelerating in an exclusive lane will narrow the speed difference between U-turning and through vehicles through a long segment to merge with through vehicles. Traffic conflicts, travel times, delays and the number of stops can be significantly reduced via the channelization of U-turning and through vehicles.

**Fig 2:**
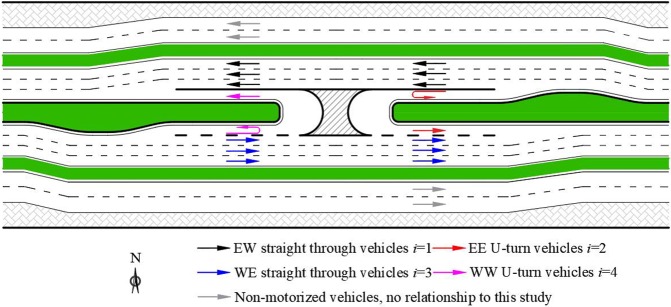
Illustration of ESUL design on provincial trunk highway. W:West, E:East, N:North, S:South.

In this study, a modified U-turn design named the exclusive spur dike U-turn lane (ESUL) is proposed to relax the traffic chaos when diverging and merging between U-turn flows and straight-traveling flows. The configuration of the ESUL design is shown in Fig 2. With the spur dike, an exclusive U-turn lane could be established, which provides a U-turn movement separate from the through flow. Diversion, deceleration, U-turn, acceleration and merge can all be performed on the ESUL without influencing the through flow. The waiting queue when too many U-turning vehicles are waiting to turn will be eliminated. The traffic conflict when U-turning vehicles want to merge is also eliminated.

The primary objective of this study is to evaluate the operational effects of ESUL. The travel time, delay and number of stops with ESUL were evaluated for various traffic situations. The traffic operations with MUTI for the same location were evaluated. The remainder of this article is organized as follows. Section 2 introduces the model development and calculation of operational measures. Data collection and calibration of the simulation model are introduced in section 3. Section 4 discusses the results of the VISSIM simulation and the sensitivity analysis of operational performance. Section 5 concludes the paper.

## Model development

### Problem description

The largest problem with the previous U-turn design studies is that the two-way straight traffic flow and the U-turn traffic flow are not separately channelized. The inner lane mixes with the through and U-turn flows. They only provide various U-turn openings, but the U-turning vehicles need to wait to merge, and the waiting queue may be longer than U-turn opening when too many vehicles need to make a U-turn. Thus, turning vehicles and straight-through vehicles in the innermost lane will cause traffic problems, such as delays, stops, lane changes and/or accidents.

#### General design

An illustration of the general design of ESUL for a U-turn median opening is shown in Fig 2.

In Fig 2, WENS are the four cardinal directions. The figure shows a 6-lane loop road in an urban area with a two-direction U-turn median opening. Two non-motorized lanes are set on both sides of the way with a 1.2-m-wide greenbelt dividing the main lanes. Flow *i* = 1 is the through vehicles from east to west. Flow *i* = 2 is the vehicles from the east and U-turn back to the east. Flow *i* = 3 is the through vehicles from west to east. Flow *i* = 4 is the U-turning vehicles from the west and back to the west through this median opening. The two through flows are high-speed flows. U-turning vehicles, flows *i* = 2, 4, always need to wait at the median opening and must yield to flow *i* = 1 in theory and flow *i* = 3 to seek an acceptable headway gap. The waiting time will be very long when the traffic volume is large, and flows *i* = 2, 4 will be forced to merge and cause a traffic conflict.

The inner two lanes of ESUL in Fig 2 are the lanes for U-turning vehicles to diverge, decelerate, U-turn, accelerate, seek the headway gap and merge into the through flows. The spur-dike sections in Fig 2 are the most significant parts that differ from other U-turn designs. The spur-dike forces the traffic flows to move outward slightly with a one-lane width. Separating the turning flow and the through flow is the core of this design, which could reduce travel times, traffic delays and the number of stops. With ESUL, flow *i* = 2 and flow *i* = 4 will not influence flow *i* = 1 and flow *i* = 3, respectively. The U-turn flows will diverge and merge with through vehicles at the operating speed.

Compared with traditional U-turn designs, the core of ESUL is the spur dike. The spur dike consists of three differences. The first one is making the whole lanes move outward slightly. The U-turning vehicles need a specific lane for U-turns to avoid the influence of the through traffic. If the spur dike is replaced by markings on the ground, drivers may not flow the markings, and they may cross the markings to drive straight [35, 36]. Second, when demands for U-turns in two directions exist, the two spur dikes in both directions could provide deceleration diversion and acceleration lanes on both sides, making maximum use of the land. Third, some U-turn designs narrow the median width to locate the U-turn lane, and the median width may be less than U-turn radius. With the spur dike, the median can easily be widened per the requirements. ESUL with a spur dike could be adjusted to different road layouts.

#### Geometry

**Fig 3:**
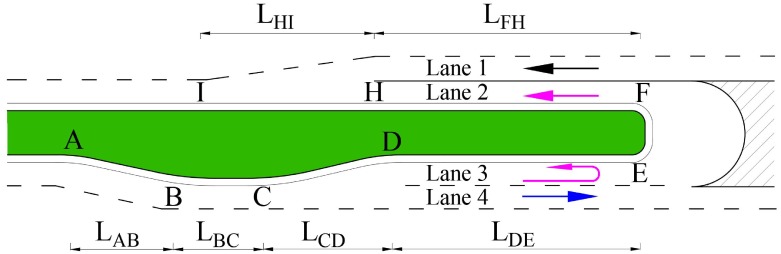
Illustration of ESUL design.

The two ESULs are mirror designs, as shown in Fig 2; only the left half of ESUL is discussed in this section. Geometric dimensioning of the ESUL design with a design speed of 80 km/h is shown in Fig 3. Lane 1 and lane 4 are through lanes of flow *i* = 1 and flow *i* = 3. Lane 2 and lane 3 are the components of ESUL for flow *i* = 4. All lengths are based on AASHTO ‘Highway Capacity Manual’ [37] and ‘A policy on geometric design of highways and streets’ [38] and on previous studies [39, 40]. Section *AB* is based on a road alignment process, section *BC* is calculated by drivers’ reaction times and movement procedures, section *CD* is the diversion part, section *DE* includes deceleration and safety distance, and section *EF* is made for vehicles to have enough space to perform a U-turn, accelerate, seek a headway gap and perform merge movement contained in sections *FH* and *HI*. All parameters are described in Table 1.

**Table 1:** Geometric parameters of ESUL.

Item	Description
*L*_*AB*_	166m. Length for all flows moved outward slightly
*L*_*BC*_	185m. Length for flow *i* = 4 recognize the U-turn sign and take action
*L*_*CD*_	50m. Diversion length to separate flow *i* = 3 and flow *i* = 4
*L*_*DE*_	42m. Flow *i* = 4 deceleration length
*L*_*EF*_	Radius = 7.26m. U-turn width, for passenger cars
*L*_*FH*_	180m. Acceleration length of flow *i* = 4
*L*_*HI*_	140m. The length of seek a headway for flow *i* = 4 and merge into flow *i* = 1

The lengths of all different sections listed above are input into the simulation model to evaluate the ESUL performance of the U-turn median opening on a freeway with a design speed of 80 km/h.

#### Development of simulation model

Ideally, data should be collected before and after road improvement (e.g., before and after the application of MUTI and ESUL) for a comparative analysis. This is difficult to do in practice, especially when the ESUL is not actually built. Therefore, it is necessary to use traffic simulation software to evaluate the changes in the traffic operating state before and after implementing the designed improvement. The VISSIM simulation software has been applied in the field of traffic simulation for more than 40 years [41]. Some achievements have been made in the research on some key parameters in traffic flow [42], including car-following model [43, 44], driving behavior model [45, 46], lane change model [46] and U-turn model [47]. The accuracy of VISSIM in traffic flow simulations is recognized [30, 48]. Therefore, in this study, both MUTI and ESUL were simulated by VISSIM for the sensitivity analysis of the operational measures for comparison.

To guarantee the accuracy of the simulation, the road geometric parameters input into VISSIM must be emphasized. In addition to the geometric length of each section of ESUL mentioned above, take the map of the actual median opening as the base map, use CAD software to draw the road, and import this map into VISSIM to guarantee that the accuracy of the geometric size and trend of the road in the simulation process are consistent with the actual situation. In this study, U-turning vehicles must yield to through vehicles. U-turning vehicles need to wait for an acceptable headway gap to cross the street.

### Calculation of operational measures

Three indices are considered and calculated to evaluate the MUTI and ESUL operational measures: travel time, delay and number of stops.

**Travel time** Travel time means the average time that it takes for all vehicles to travel a given distance. In the VISSIM simulation, “it consists of a from section and a to section. The mean travel time from traversing the from section up to traversing the to section, including the waiting time and/or holding time, is calculated as well as the distance traveled between the start section and destination section” [48]. Travel time can be calculated as shown in Eq 1

$$\begin{array}{c} T_{{}_{i}}^{E}=\frac{\sum_{j=1}^{Q_{i}^{E}}t_{{}_{ij}}^{E}}{Q_{i}^{E}}\;\forall i=1,2,3,4 \end{array}$$ (1)

where $T_{{}_{i}}^{E}$ denotes the travel time of each traffic flow with the ESUL design. $t_{{}_{ij}}^{E}$ denotes the travel time of a single vehicle in each flow with the ESUL design, and $Q_{i}^{E}$ denotes the total number of vehicles that passed in each flow with the ESUL design.

$$\begin{array}{c} T_{{}_{i}}^{M}=\frac{\sum_{j=1}^{Q_{i}^{M}}t_{{}_{ij}}^{M}}{Q_{i}^{M}}\;\forall i=1,2,3,4 \end{array}$$ (2)

where $T_{{}_{i}}^{M}$ denotes the travel time of each traffic flow with the MUTI design. $t_{{}_{ij}}^{M}$ denotes the travel time of a single vehicle in each flow with the MUTI design, and $Q_{i}^{M}$ denotes the total number of vehicles that passed in each flow with the MUTI design.

**Delay** Delay means the difference between the actual travel time and the driver’s expected travel time. The reasons for the difference include traffic interference, traffic management and control measures. In this study, delay includes stop delay and travel delay.

$$\begin{array}{c} D_{i}^{E}=d_{i1}^{E}+d_{i2}^{E}\;\forall i=1,2,3,4 \end{array}$$ (3)

where $D_{i}^{E}$ denotes the total delay of each flow with the ESUL design. $d_{i1}^{E}$ denotes the stop delay, and $d_{i2}^{E}$ denotes the travel delay of each flow with the ESUL design.

$$\begin{array}{c} D_{i}^{M}=d_{i1}^{M}+d_{i2}^{M}\;\forall i=1,2,3,4 \end{array}$$ (4)

where $D_{i}^{M}$ denotes the total delay of each flow with the MUTI design. $d_{i1}^{M}$ denotes the stop delay, and $d_{i2}^{M}$ denotes the travel delay of each flow with the MUTI design.

**Number of stops** In the VISSIM simulation, it means all situations in which a vehicle comes to a stop (speed = 0), except for stops at public transport stops and in parking lots [48]. The number of stops can be calculated by the total number of stops of each flow divided by the total number of vehicles in this flow.

$$\begin{array}{c} S_{{}_{i}}^{E}=\frac{\sum_{j=1}^{Q_{i}^{E}}s_{{}_{ij}}^{E}}{Q_{i}^{E}}\;\forall i=1,2,3,4 \end{array}$$ (5)

where $S_{{}_{i}}^{E}$ denotes the average number of stops of each flow with the ESUL design. $s_{{}_{ij}}^{E}$ denotes the number of stops of each vehicle, and $Q_{i}^{E}$ denotes the total number of vehicles in this flow with the ESUL design.

$$\begin{array}{c} S_{{}_{i}}^{M}=\frac{\sum_{j=1}^{Q_{i}^{M}}s_{{}_{ij}}^{M}}{Q_{i}^{M}}\;\forall i=1,2,3,4 \end{array}$$ (6)

where $S_{{}_{i}}^{M}$ denotes the average number of stops of each flow with the MUTI design. $s_{{}_{ij}}^{M}$ denotes the number of stops of each vehicle, and $Q_{i}^{M}$ denotes the total number of vehicles in this flow with the MUTI design.

## Data

### Data collection

Real traffic data were collected to build and calibrate the simulation model in VISSIM. The location for data collection needs to reflect the characteristics of the traffic flow in the problem statement, such as median opening for U-turn vehicles, high volume, far from upstream and/or downstream signalized intersection and good sight distance. Data from a U-turn median opening that satisfies the above conditions were collected in Xi’an City (Shaanxi Province, China) because Xi’an was ranked as the second-most congested city in China in the second season of 2018 [49].

**Fig 4:**
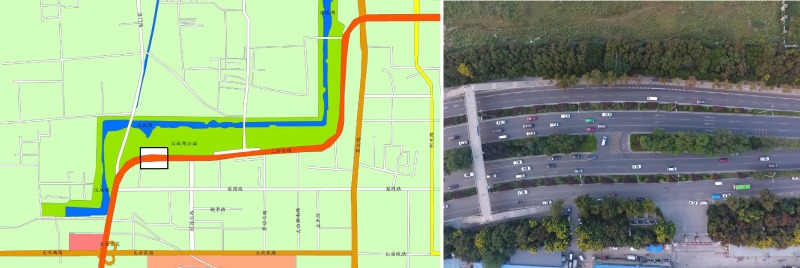
A median opening at northwest corner 2nd loop road in Xi’an. Coordinates:108.903898,34.301482. a:The U-turn median opening location schematic. b: The present median opening graphic took by a drone at height of 150m.

Fig 4 shows a typical MUTI median opening in the northwest corner of the 2nd loop road in Xi’an City. The loop road is a 6-lane and access control loop road with a speed limit of 80 km/h. The lane width of each lane is 3.5 m. The median width is 1.2 m on average and 10 m at the U-turn median opening. The length of the opening is 17 m. Additionally, 9-m-wide non-motor-vehicle lanes are set on both sides with a 1.5m-wide greenbelt to divide them from the main lanes. Both directions have a U-turn demand in this U-turn median opening. The field studies did not involve endangered or protected species. The city for data collection has a population of over 9.9 million, area of 10,752 square kilometers and over 3.5 million vehicles.

The reason why we set a U-turn median opening here is the distance between the upstream and downstream interchanges is 5.1 km. No entrance or exit exists in this section, and the running speed could reach the speed limit after passing the median opening 200 m. The distance from the median opening to the upstream interchanges is 1.4 km, and it is 3.6 km to the downstream. Clearly, the non-viaduct form of the urban loop road splits the communities on both sides of the road [50]. Specifically, when vehicles want to turn left, they need to travel to the next interchange or intersection to make a left turn. Under the circumstance where an interchange is not built for various reasons and the distance between the two interchanges is relatively long, it is feasible to realize the left-turning vehicles turning through the median opening. If no U-turn median opening is designed, then U-turning vehicles would detour 10 km and 9 min at most. In fact, the U-turn median opening plays the role of an interchange or intersection. Both directions have U-turning vehicles, and WW U-turning vehicles (flow *i* = 4) outnumber EE U-turning vehicles (flow *i* = 4). When there are no U-turning vehicles, flows *i* = 1, 3 operate from 45 km/h∼55 km/h in the inner lane. If flows *i* = 2, 4 have one or more vehicles, then flows *i* = 1, 3 in the inner lane would slow to 20 km/h or even stop for the queue. U-turning vehicles must yield to through vehicles and wait for headway gaps, but U-turning vehicles will be forced to merge if the waiting time is too long, and this movement will cause the opposite straight-running vehicles to slow or even stop. If flows *i* = 2, 4 have to wait in a queue, through vehicles (flows *i* = 1, 3) will change to the middle lane, and they may even be forced to change lanes and make the middle lane vehicles slow or even stop.

A pedestrian bridge is located 70 m west of the median opening (Fig 4). Two video cameras were placed on this bridge to take videos for determining the number of vehicles, and two radars were used to collect two-direction traffic flow data, including the trajectories and speeds of vehicles. The criteria of the collected data are as follows:

- Collect all vehicle speed during the collect period.
- Collect all vehicle types during the collect period.
- Collect all vehicle trajectories.

**Fig 5:**
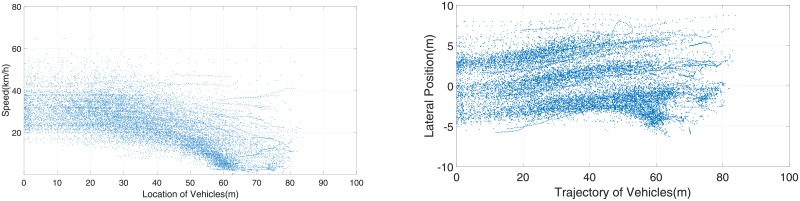
Speed and trajectories of east to west flows. a:Speed of vehicles from East to West. b: Trajectories of vehicles from East to West.

**Fig 6:**
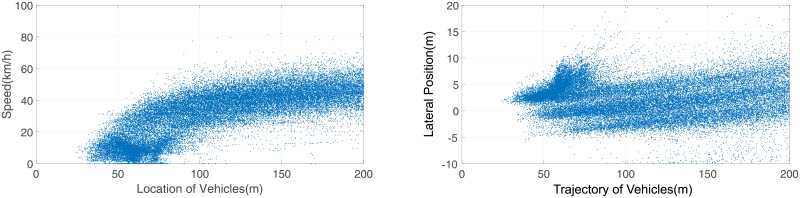
Speed and trajectories of west to east vehicles. a:Speed of vehicles from West to East. b: Trajectories of vehicles from West to East.

The preliminary radar data need to be processed to illustrate the vehicle speeds and trajectories. Discontinuous data were eliminated from the preliminary radar data. In general, data within 200 m could be guaranteed to have effective accuracy. However, in the direction from east to west, flow *i* = 1 and flow *i* = 2 always wait and stop before the U-turn median opening, which made the radar unable to distinguish each vehicle, and the final data are only effective within 90 m. The collected data are presented below in Table 2, Figs 5 and 6.

**Table 2:** Collect vehicle information of the investigation.

Item	Valley Hour (13:00∼14:00)				Peak Hour(18:00∼19:00)			
Direction	East to West		West to East		East to West		West to East	
*Flow*	*i* = 1	*i* = 2	*i* = 3	*i* = 4	*i* = 1	*i* = 2	*i* = 3	*i* = 4
*Car*	3058	53	2920	424	3640	182	3040	396
*Truck*/*Bus*	36	1	64	22	86	2	46	14
*Averagespeed*(*km*/*h*)	37.5	7.6	11.8	35.8	32.67	6.5	10.7	33.26
*Max*.*speed*(*km*/*h*)	67.5	24.3	23.2	81.6	68.5	15.3	13.2	64.3
*Min*.*speed*(*km*/*h*)	5.8	0	0	9.2	0	0	0	0

Minimum speed is 0km/h means some vehicles were stop and wait to move.

The speeds of all vehicles from east to west are shown in Fig 5a. The horizontal coordinate 70 m is the U-turn location, and the traffic flow operates from the right to left of the figure. It is clear that the majority of vehicles operate at a low speed near the U-turn section and then accelerate after the U-turn section. U-turning vehicles (flow *i* = 4) strongly affected the through vehicles (flow *i* = 1). Few lines in the figure changed gently, indicating that the flow *i* = 1 vehicles did not decelerate too much, which represents free flow status. The peak points of all lines in Fig 5a were under 80 km/h and mainly centralized between 20∼40 km/h. The operating speed was much lower than the speed limit.

Fig 5b shows the trajectories of vehicles from east to west. It is not difficult to distinguish 3 lanes and U-turning vehicle trajectories. The trajectories correspond to the geometry of road alignment. The color of the lowest trajectory was much deeper and wider than that of the other two trajectories, which means that the U-turn flow *i* = 4 mixed with the through flow *i* = 1, and the two flows drive together in the inner lane from coordinate 60∼40 m. No obvious trajectory changes from the inner lane to the middle lane during 60∼40m showed that it is not easy to change to the lane due to large traffic volume. Thus, the U-turn flow *i* = 4 mostly affected the inner lane flow.

Fig 6a shows the speeds of vehicles from west to east. The overall speed is higher than that of east to west, and the speed was still higher even for the same length after the WW U-turn section (coordinate 70 m∼130 m). This result indicates that WW U-turning vehicles (flow *i* = 4) have a lower influence on through flow *i* = 3 due to the diversion movement than merge movement in Fig 5. However, many lines in Fig 6a started from 0 km/h, which means that the U-turning vehicles still caused deceleration and stops for the entire traffic flow.

Fig 6b shows the 3 lanes and U-turning vehicle trajectories from west to east. The U-turn section has the deepest color. All the U-turn movement data cannot be collected because trees were planted in the median strip (Fig 4b), which blocked the radar signal. Only the turning action could be captured, as shown in Figs 5b and 6b.

**Fig 7:**
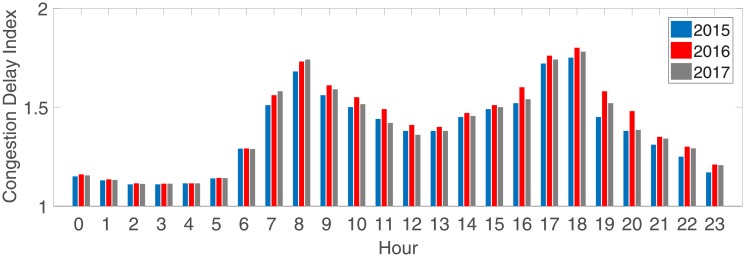
The 24-hour congestion trend of major cities from 2015 to 2017 [51]. The morning peak appeared from 07:00∼09:00, the evening peak appeared from 17:00∼19:00, and the valley, excluding late night, appeared from 12:00∼14:00.

**Fig 8:**
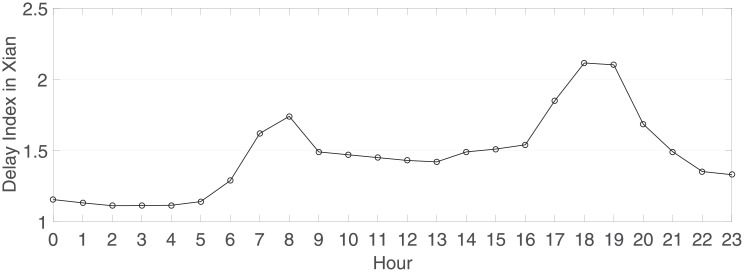
The 24-hour congestion delay index for Xi’an on 2018.09.12 [51, 53]. The peak appeared from 18:00∼19:00, and the valley, excluding late night, appeared from 13:00∼14:00.

Real data collection requires both valley hour data and peak hour data, according to the *2017 Traffic Analysis Reports for Major Cities in China* proposed by AutoNavi Traffic big data [51]. AutoNavi is a Chinese web mapping, navigation and location-based services provider [52]. In the report, the 24-hour congestion trend of major cities from 2015 to 2017 is as shown in Fig 7:

The report [51, 53] also provides a real-time 24-hour congestion delay index for 100 cities in China. We collected the delay index data of Xi’an for a day (2018.09.12) as shown in Fig 8:

The data were collected from 13:00∼14:00 (valley hour) and from 18:00∼19:00 (peak hour). During the investigation, only private cars and a few large trucks and buses passed this section. Thus, the classification of vehicle types was easy: private cars are denoted as Car, and all types of trucks and buses are denoted as Truck/Bus in the collected data. The collected data are shown in Table 2.

During the investigation, ideally, the U-turning vehicles must yield to through vehicles according to the traffic rules. However, except when the U-turning vehicles could easily merge when the headway was sufficient, many U-turning vehicles were forced to merge with through vehicles and made the through vehicles suddenly stop due to U-turning vehicles being unable to wait too long (approximately 20 s according to the investigation). The numbers of forced merge times and vehicles are as Table 3:

**Table 3:** Number of force merge times.

Item	Valley Hour (13:00∼14:00)		Peak Hour(18:00∼19:00)	
Direction	EE U-turn	WW U-turn	EE U-turn	WW U-turn
Number of force merge times	8	96	23	92
Number of force merge U-turn vehicles	19	175	34	187
Number of total U-turn vehicles	54	446	184	410
Ratio of force U-turn vehicles (%)	35.19	39.24	18.48	45.61

A forced merge means that through vehicles were forced decelerate to under 20 km/h from their operating speed and even stopped to wait for U-turning vehicles to merge during the U-turn segment. The following U-turning vehicles may merge one by one before the through vehicles restarted, and these U-turning vehicles ranged to the number of forced merge U-turning vehicles.

The collected data show the following characteristics:

1. The operating speed of flows *i* = 1, 3 was much lower than the speed limit and design speed (80 km/h)
2. U-turning vehicles (flows *i* = 2, 4) had a low speed and needed to wait during the merge movement
3. A large number of U-turning vehicles were not willing to wait long, and they forced a merge, which caused serious delays and stops for through vehicles

### Calibration of VISSIM simulation model

The VISSIM parameters need to be calibrated and validated with the investigation data to ensure that the simulation is accurate [54, 55]. Traffic data, capacity and geometric measures were collected during the investigation to calibrate the simulation model in VISSIM [56]. In this study, the collected data contained valley and peak hour data; thus, both situations need to be calibrated. The data collected above were considered as the inputs in VISSIM:

1. Total vehicle volume of cars and trucks/buses in each direction
2. Ratio of cars and trucks/buses in each direction
3. Ratio of U-turning and through cars and trucks/buses separately
4. Expected speed of each flow
5. All above data were input into VISSIM with valley hour and peak hour separately

Several calibrated parameters in the VISSIM simulation model are the gap-accepting model, car-following model and lane-changing model [57, 58]. Capacity is mainly used for VISSIM simulation model calibration [13]. The capacity could reflect multiple properties of the model, and it is the most sensitive to route choice behavior. If the capacity of the VISSIM model is similar to the collected data, then the VISSIM model accuracy could be confirmed, and the VISSIM model could be used to evaluate the improvement of the design for traffic flow [59]. The capacity can be calculated by Eq 7:

$$\begin{array}{c} C=\frac{3600}{\bar{h_{t}}} \end{array}$$ (7)

where *C* denotes the ideal capacity (veh/h). $\bar{h_{t}}$ denotes the average minimum headway (s). The capacity used to estimate the simulation error is the Mean Absolute Percent Error (MAPE), which can be calculated as Eq 8:

$$\begin{array}{c} MAPE=\frac{1}{n}\sum_{i=1}^{n}|\frac{C_{v}^{i}-C_{f}^{i}}{C_{f}^{i}}| \end{array}$$ (8)

where *n* denotes the 4 different flows in this study, $C_{v}^{i}$ is the capacity simulated in the VISSIM model (veh/h), and $C_{f}^{i}$ is the capacity of the investigation (veh/h). The calculated *MAPE* is presented in Table 4.

**Table 4:** VISSIM simulation calibration results.

Item	Valley Hour				Peak Hour			
Direction	East to West		West to East		East to West		West to East	
*Flow*	*i* = 1	*i* = 2	*i* = 3	*i* = 4	*i* = 1	*i* = 2	*i* = 3	*i* = 4
Investigated capacity (veh/h)	3094	54	2984	446	3726	184	3086	410
Simulated capacity (veh/h)	3004	58	3037	405	3521	130	3082	386
Individual *MAPE* (%)	-2.9	9.1	-9.0	1.8	-5.5	-28.9	-5.8	-0.1
*MAPE* (%)	5.7				10.1			

The capacity error scope in the VISSIM simulation model and reality is 5.7% in valley hour and 10.1% in peak hour. The estimation error can be considered acceptable in practical engineering applications [13, 60].

## Results

### Operational features with ESUL and MUTI

The VISSIM simulation models are developed for present realistic U-turn condition (MUTI) and improved U-turn design situation (ESUL) with features including travel time, delay and number of stops. These actual traffic parameters were input into the VISSIM simulation model. The simulation results in Tables 5 and 6 are based on the real data from Table 2.

**Table 5:** Operational performance of MUTI and ESUL with investigation data (valley hour).

Item	Travel Time(s)			Delay(s)			Number of Stops		
Flow	MUTI	ESUL	Rate*(%)	MUTI	ESUL	Rate*(%)	MUTI	ESUL	Rate*(%)
*i* = 1	62.13	60.54	-2.56	18.37	17.51	-4.68	0.09	0.03	-66.67
*i* = 2	80.97	59.16	-26.94	26.87	8.95	-66.70	0.78	0	-100
*i* = 3	69.64	68.9	-1.06	19.77	18.43	-6.78	0.09	0.03	-66.67
*i* = 4	77.07	68.39	-11.26	27.99	19.17	-31.51	0.74	0.28	-62.16

*Rate = (ESUL-MUTI)/MUTI*100%.

**Table 6:** Operational performance of MUTI and ESUL with investigation data (peak hour).

Item	Travel Time(s)			Delay(s)			Number of Stops		
Flow	MUTI	ESUL	Rate*(%)	MUTI	ESUL	Rate*(%)	MUTI	ESUL	Rate*(%)
*i* = 1	67.61	64.43	-4.70	23.55	21.19	-10.02	0.37	0.07	-81.08
*i* = 2	87.17	62.71	-28.06	33.60	13.11	-60.98	0.95	0.15	-84.21
*i* = 3	74.39	68.8	-7.51	24.39	17.92	-26.53	0.38	0.03	-92.11
*i* = 4	98.67	69.91	-29.15	50.63	21.12	-58.29	3.36	0.29	-91.37

*Rate = (ESUL-MUTI)/MUTI*100%.

The ESUL design in this article effectively improved the performances of all vehicles except for flow *i* = 2 and flow *i* = 4.

In the direction from west to east, for flow *i* = 2 and flow *i* = 4, the travel time and delay both increased. The travel time slightly changed, 3.59% and 2.20%, but it did not have too much influence on vehicles. The delay increased 25.29% and 5.33%, but the growth value was only 0.43 s and 0.12 s. The number of stops had no change.

For vehicles from east to west, for flow *i* = 1 and flow *i* = 3, the travel time decreased 13.76% and 37.57%, the delay decreased 33.33% and 78.83%, and the number of stops decreased 100% and 82.64%. For flow *i* = 5 and flow *i* = 6, both three indices obviously decreased, from 7.84% to 73.84%. The operating situations of all four of these flows greatly improved with the ESUL. Moreover, the price is a slight increase for flow *i* = 2 and flow *i* = 4.

The above results show that the ESUL design in this article has considerable potential to effectively improve the performance compared to MUTI. The ESUL design is suitable for urban roads in terms of travel time, delay and number of stops.

### Safety evaluation with ESUL and MUTI

Both operation evaluation and safety evaluation should be included in a complete evaluation of a traffic entity. The surrogate safety assessment model (SSAM) is widely used to extract traffic conflicts from VISSIM simulation trajectories. SSAM is a technique combining microsimulation and automated conflict analysis that analyzes the frequency and character of narrowly avoided vehicle-to-vehicle collisions in traffic to assess the safety of traffic facilities without waiting for a statistically above-normal number of crashes and injuries to occur [61, 62].

Table 2 data were used in VISSIM simulation to evaluate the safety condition between ESUL and MUTI under both valley and peak hours. The thresholds of simulated conflicts in terms of TTC (time-to-collision), PET (post-encroachment time), rear-end angle and crossing angle were set as 1.5 s, 5.0 s, 30.00° and 80.00°, respectively, according to [63].

The safety evaluation results of ESUL and MUTI during the valley and peak hours are shown as Tables 7 and 8, respectively.

**Table 7:** Safety performance of MUTI and ESUL with investigation data (valley hour).

Item	Crossing	RearEnd	Lane Change	Total
MUTI	78	33	79	190
ESUL	0	129	4	133

**Table 8:** Safety performance of MUTI and ESUL with investigation data (peak hour).

Item	Crossing	RearEnd	Lane Change	Total
MUTI	78	32	82	192
ESUL	0	141	7	148

The results of the safety evaluation showed that compared to MUTI, ESUL could eliminate crossing conflicts and drastically reduce lane change conflicts. While rear end conflicts increased significantly, total conflicts were reduced, which could indicate that compared to MUTI, ESUL also performs better in terms of safety evaluation.

### Sensitivity analysis of operational performance

All possible traffic situations cannot be included in the collected data, which restricted the simulation and evaluation of sustainable traffic operation in this article. Different traffic situation combinations were specified in the VISSIM simulation model to ensure that more possible situations were covered between MUTI and ESUL.

Some parameters need to be input into VISSIM during the sensitivity analysis, including car/truck (bus) ratio, through/U-turn ratio and traffic volume changes. Peak hour parameters were selected in the sensitivity analysis. The traffic volume change depends on V/C and when the design speed is 80 km/h. The traffic volume is 6930 veh/h for urban 3-lane freeway segment corresponding to service level E according to [37]. The sensitivity analysis ranges from 0.2∼1.0 V/C with an increase of 693 veh/h (0.1 V/C). The U-turning vehicles ratio ranges from 1.72% to 13% based on Table 2, and the U-turn ratio range was 0.03∼0.15 in sensitivity analysis with an increase of 0.03. The parameters are as Table 9:

**Table 9:** Parameters input into sensitivity analysis in VISSIM.

Item	Value								
Car/Truck(bus) ratio	3822:88 (EW)/3436:60 (WE)								
U-turn ratio (%)	0.03/0.06/0.09/0.12/0.15								
V/C	0.2	0.3	0.4	0.5	0.6	0.7	0.8	0.9	1.0
Volume (veh/h)	1386	2079	2772	3465	4158	4851	5544	6237	6930

**Fig 9:**
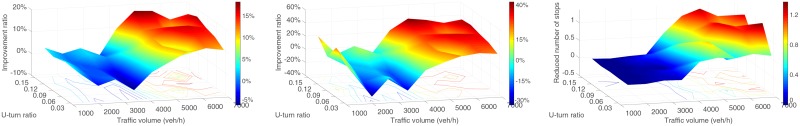
Sensitivity analysis of flow 1, EW through vehicles. The X axis is different traffic volumes, the Y axis is u-turn ratio and the Z axis is improvement ratio (Ratio = (MUTI-ESUL)/MUTI*100%) in a,b, and reduced times (Reduced times = MUTI-ESUL) in c. a. Travel time improvement ratio. b. Delay improvement ratio. c. Reduced number of stops.

**Fig 10:**
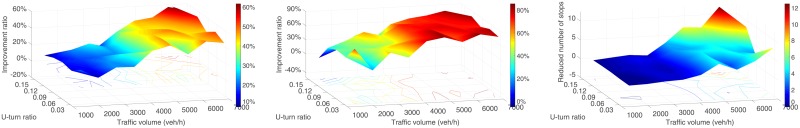
Sensitivity analysis of flow 2, EE U-turn vehicles. The X axis is different traffic volumes, the Y axis is U-turn ratio and the Z axis is improvement ratio (Ratio = (MUTI-ESUL)/MUTI*100%) in a,b, and reduced times (Reduced times = MUTI-ESUL) in c. a. Travel time improvement ratio. b. Delay improvement ratio. c. Reduced number of stops.

**Fig 11:**
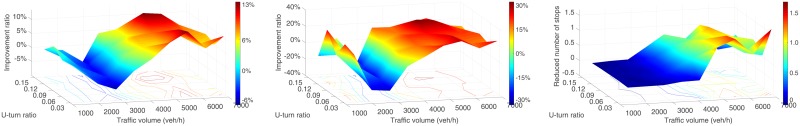
Sensitivity analysis of flow 3, WE through vehicles. The X axis is different traffic volumes, the Y axis is U-turn ratio and the Z axis is improvement ratio (Ratio = (MUTI-ESUL)/MUTI*100%) in a,b, and reduced times (Reduced times = MUTI-ESUL) in c. a. Travel time improvement ratio. b. Delay improvement ratio. c. Reduced number of stops.

**Fig 12:**
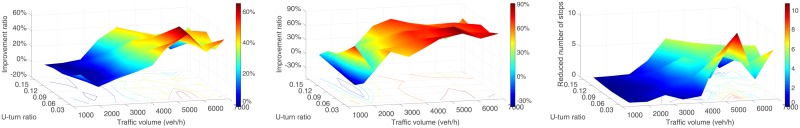
Sensitivity analysis of flow 4, WW U-turn vehicles. The X axis is different traffic volumes, the Y axis is u-turn ratio and the Z axis is improvement ratio (Ratio = (MUTI-ESUL)/MUTI*100%) in a,b, and reduced times (Reduced times = MUTI-ESUL) in c. a. Travel time improvement ratio. b. Delay improvement ratio. c. Reduced number of stops.

The sensitivity analysis results were evaluated with three indices: travel time, delay and number of stops. We simulated different U-turn ratios and V/C under ESUL and MUTI. The results of travel time and delay showed an improvement in the ratio between ESUL and MUTI: Ratio = (MUTI-ESUL)/MUTI*100%. The result of the number of stops means a reduced number of stops between two designs: Reduced times = MUTI-ESUL. The positive values in the sensitivity results mean that ESUL improved the traffic operating situations, and negative values represent the opposite meaning. The sensitivity results of the four directions are shown in Figs 9–12.

The sensitivity analysis of flow *i* = 1 (EW through vehicles) is shown in Fig 9. Fig 9a shows that the travel time ratio decreased with ESUL, the travel time increased within 10% when the traffic volume was less than 4000 veh/h and decreased within 0%∼20% when the traffic volume was more than 4000 veh/h. The delay increased when the traffic volume was under 4000 veh/h and decreased when the traffic volume was beyond 4000 veh/h in Fig 9b. The peak value that appeared at the U-turn ratio was 0.15, and the traffic volume was 4000 veh/h and reached more than 40%. The number of stops also had the same trend, and when the traffic volume was under 4000 veh/h, the number of stops had almost no change. When the traffic volume exceeded 4000 veh/h, the reduction in the number of stops increased, and 1.3 was the peak value in Fig 9c.

Fig 10 shows the sensitivity results of flow *i* = 2 (EE U-turning vehicles). All three indices of flow *i* = 2 greatly improved. The travel time shown in Fig 10a decreased from 0%∼60% as the traffic volume increased, and the improvement became more obvious when the traffic volume exceeded 3000 veh/h. The delay of flow *i* = 2 reduced much more than the travel time and reached 80% as the peak value (Fig 10b). The number of stops also decreased as the traffic volume increased. For flow *i* = 2, the reduced number of stops was mainly between 0∼12 times, which was a great improvement (Fig 10c). The peak value appeared when the traffic volume was 6237 veh/h and U-turn ratio was 0.15.

The sensitivity analysis results of flow *i* = 3 (WE through vehicles) are shown in Fig 11. One clear ditch appeared in both travel time (Fig 11a) and delay (Fig 11b). When the traffic volume was approximately 2772 veh/h (0.4 V/C), the travel time of flow *i* = 3 increased approximately 6% and then decreased as the traffic volume increased. However, when the traffic volume was between 3000∼4000 veh/h, the travel time improved back to 0%. The delay of flow *i* = 3 had the same trend as travel time. The delay of flow *i* = 3 increased when the traffic volume was approximately 2772 veh/h by approximately 30% and decreased 30% most when the traffic volume was approximately 4851∼5544 veh/h. The number of stops had no change when the traffic volume was under 4000 veh/h and reduced after that, and the peak value of 1.5 appeared when the traffic volume was 6930 veh/h.

Flow *i* = 4 (WW u-turn vehicles) sensitivity analysis results are shown in Fig 12. The travel time decreased 0%∼60% as the traffic volume increased. The delay increased 30% when the traffic volume was 2079 veh/h and decreased up to 90% under other traffic volumes. The number of stops decreased when the traffic volume was greater than 4000 veh/h and reached the peak value of 10 when the traffic volume was 5544 veh/h and U-turn ratio was 0.03, which was a significant improvement for flow *i* = 4.

The sensitivity analysis results shown in Figs 9–12 showed the following characteristics:

1. Traffic volume had considerably more influence on the performance of ESUL than U-turn ratio
2. U-turn ratio had an effect on the performance of ESUL, but the effect was not obvious
3. Flows *i* = 1, 3 and flows *i* = 2, 4 have similar trends of ESUL performance separately
4. When the traffic volume was under 3000 veh/h, ESUL has no obvious performance and even a negative improvement
5. Traffic volume 4000 veh/h was a watershed, and ESUL played a significant role when the traffic volume was greater than 4000 veh/h
6. ESUL could improve traffic operating situations in 4 directions, especially for U-turn flows

In summary, according to the results from the sensitivity analysis, it can be found that ESUL could be a good design for improving sustainable traffic operating situations on U-turn median openings. ESUL could reduce travel time, delay and number of stops of traffic flows in all four directions. The improvement of ESUL is obvious, especially when the traffic volume is higher than 4000 veh/h. Traffic conflicts caused by U-turn maneuvers near the median opening vanished with ESUL. ESUL is good design compared with MUTI.

**Fig 13:**
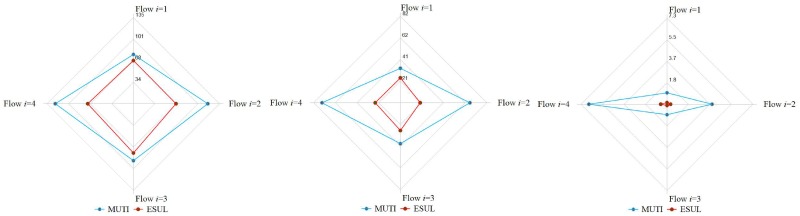
Effectiveness of ESUL compared with MUTI. Traffic volume is 4851 veh/h and U-turn ratio is 0.12. a.Travel time. b.Delay. c.Number of stops.

A comparison of the operational effect between MUTI and ESUL was also conducted. Based on Figs 9–12, when the traffic volume is greater than 4000 veh/h, ESUL has a better performance than MUTI in terms of lower travel times, delays and number of stops. Fig 13 presents the comparison between MUTI and ESUL when the traffic volume is 4851 veh/h and U-turn ratio is 0.12. The vehicle travel times, delays and number of stops for ESUL are all much lower than MUTI under flows in all four directions, which means that the ESUL has a better operational performance than MUTI.

## Conclusions

Traditional MUTI has the disadvantage that it will cause traffic conflicts when U-turning vehicles wait in a long queue or merge with through traffic. In this study, a modified U-turn design, named the exclusive spur dike U-turn lane (ESUL), is proposed. The core design of ESUL is that two spur dikes were set on both sides of the road and exclusive U-turn lanes could be established, which provides U-turn movement separate from the through flow. Diversion, deceleration, U-turn, acceleration and merge can all be done on the ESUL without influencing the through flow. Traffic conflicts caused by U-turning vehicles queuing and/or merging with through traffic will be significantly reduced.

The results show that ESUL has obvious improvements over MUTI with large traffic volumes. In this case, the travel time is reduced by 13% to 60%, vehicle delay is decreased by 30% to 90%, and the number of stops is decreased by 1.5 to 12 times. When the traffic volume is lower than 3000 veh/h, the ESUL has very little improvement compared to MUTI, but it at least has a similar result. As vehicle ownership continues to increase, the ESUL is the most suitable measure to cope with high traffic volume and U-turn ratio.

The findings of this study can be useful in reducing traffic conflicts (including delays, stops and potential accidents) caused by U-turning vehicles, which could be utilized as a guideline for road designers and municipal construction departments to evaluate and determine the design of or reconstruct the ESUL. Before ESUL is used in the future, some issues can be studied further: first, whether the ESUL geometry size could be reduced while keeping the operational features unchanged; second, the distance between the ESUL location and signalized intersection should be studied when ESUL implemented in urban streets. The authors recommend that future studies could focus on these issues.
